# Cumulative barriers to retention in methadone treatment among adults from rural and small urban communities

**DOI:** 10.1186/s13722-022-00316-3

**Published:** 2022-07-15

**Authors:** Emily Pasman, Rachel Kollin, Michael Broman, Guijin Lee, Elizabeth Agius, Jamey J. Lister, Suzanne Brown, Stella M. Resko

**Affiliations:** 1 School of Social Work, Wayne State University, 5447 Woodward Ave, Detroit, MI 48202 USA; 2 Center for Behavioral Health and Justice, Wayne State University, 5201 Cass Ave, Detroit, MI 48202 USA; 3 School of Social Work, Rutgers University, 120 Albany Street, Tower One, New Brunswick, NJ 08901 USA; 4 Merrill Palmer Skillman Institute, Wayne State University, 71 E Ferry St, Detroit, MI 48202 USA

**Keywords:** Methadone, Barriers, Rural, Opioids, Treatment retention

## Abstract

**Background:**

Though methadone has been shown to effectively treat opioid use disorder, many barriers prevent individuals from accessing and maintaining treatment. Barriers are prevalent in less populated areas where treatment options are limited. This study examines barriers to retention in methadone treatment in a small Midwest community and identifies factors associated with greater endorsement of barriers.

**Methods:**

Patients at an opioid treatment program (N = 267) were recruited to complete a computer-based survey onsite. Surveys assessed demographics, opioid misuse, depression and anxiety symptoms, trauma history and symptoms, social support, and barriers to retention in treatment (e.g., childcare, work, housing, transportation, legal obligations, cost, health). Descriptive statistics were used to examine individual barriers and multiple regression was calculated to identify demographic and psychosocial factors associated with greater cumulative barriers.

**Results:**

Most participants reported at least one barrier to retention in treatment and more than half reported multiple barriers. Travel hardships and work conflicts were the most highly endorsed barriers. Past year return to use (*B* = 2.31, *p* = 0.004) and more severe mental health symptomology (*B* = 0.20, *p* = 0.038) were associated with greater cumulative barriers. Greater levels of social support were associated with fewer barriers (*B* = − 0.23, *p* < 0.001).

**Conclusion:**

This study adds to the limited research on barriers to retention in methadone treatment among patients in rural and small urban communities. Findings suggest flexible regulations for dispensing methadone, co-location or care coordination, and family or peer support programs may further reduce opioid use and related harms in small communities. Individuals with past year return to use reported a greater number of barriers, highlighting the time following return to use as critical for wraparound services and support. Those with co-occurring mental health issues may be vulnerable to poor treatment outcomes, as evidenced by greater endorsement of barriers. As social support emerged as a protective factor, efforts to strengthen informal support networks should be explored as adjunctive services to methadone treatment.

## Background

Accessible treatment for opioid use disorder (OUD) continues to be an urgent need in the United States. In 2020, among the 2.5 million people aged 12 or older with a past year OUD, only 11.2% received pharmacological treatment [1]. Despite efforts to expand the availability of the three Food and Drug Administration-approved medications to treat OUD (buprenorphine, naltrexone, and methadone), accessibility gaps persist. Accessibility issues are particularly pronounced in rural communities, where opioid prescribing rates are higher [2], and rates of opioid overdose death have increased dramatically [3]. While both urban and rural communities suffer from high rates of opioid prescription and overdose deaths [4], addressing OUD in rural areas is more difficult due to limited resources for prevention, treatment, and recovery [5], as well as lack of social networks and economic opportunity disadvantage [4].

Methadone has been shown to reduce the risk of opioid overdose and better aid in treatment retention compared to nonpharmacological treatments and supports alone [6]. However, a variety of barriers prevent individuals from accessing and maintaining methadone treatment. Unlike other medications for OUD available in office-based settings, only certified opioid treatment programs (OTPs) can dispense methadone. OTPs are highly regulated by federal and state policies which require supervised medication consumption, frequent urinalysis, and counseling to accompany treatment. About half of the nation’s OTPs are for-profit organizations, and treatment at these programs is only available to those who have accepted forms of insurance or who can pay out of pocket [7]. Further, the majority of OTPs are located in large urban areas [8]. As a result, patients in less populated regions must travel further distances to receive their medication [9], often more than 50 miles and across state lines [10]. This is a particular challenge for individuals without reliable transportation or public transportation options [11]. Additionally, stigma toward methadone may be a particular problem in rural areas dominated by abstinent treatment preferences, affecting treatment availability, referrals, and uptake of MOUD [8, 12]. These regulatory, geographic, financial, and attitudinal barriers can prevent individuals from initiating treatment, and can also affect treatment retention. Though most people would benefit from long-term treatment, less than three quarters of patients are retained in methadone treatment for even 6 months [13].

Understanding the barriers individuals face in maintaining treatment is essential for treatment retention and long-term outcomes. Though few studies have examined retention barriers, specifically, research suggests certain individual characteristics may be associated with treatment retention. Male gender [14], minority race/ethnicity [15, 16], and younger age [17] have been negatively associated with OUD treatment retention. Distance from home to treatment [18–20], cost of child care [21], and insurance coverage [15] have been found to predict treatment retention as well. The presence of a co-occurring mental health condition may be another factor affecting treatment retention. Research indicates individuals with co-occurring disorders seldom receive treatment for both mental health and substance use issues [22]. For those who do access substance use treatment, mental health issues are associated with poor treatment engagement [23]. Accessibility of integrated substance use and mental health treatment is a particular challenge in rural communities due to a lack of qualified providers [11].

For methadone patients, social support may serve as a protective factor against barriers to treatment retention. The importance of social support for achieving and maintaining positive treatment outcomes is well-documented [24–26]. For patients receiving methadone treatment, the support of a partner or spouse [27] and family and close friends [28, 29] have been identified as particularly important. Greater perceived social support has been linked to the initiation of treatment and sustained recovery for individuals receiving medication for OUD [30]. Thus, interventions that include supportive friends and family members as part of OUD treatment may effectively reduce treatment barriers and improve retention [31].

Little is known about barriers to retention in methadone treatment in rural and small urban communities, or how multiple barriers accumulate. In their systematic review of rural-specific barriers to medication for OUD, Lister et al. [8] found no rural studies that analyzed barriers to methadone treatment specifically. Although this is not surprising given most OTPs are located in large urban areas, this highlights a need for research on barriers to methadone treatment in less populated areas. Further, studies in large urban areas and nation-wide studies that have examined methadone treatment retention have typically relied on administrative data rather than patients’ first-hand perspectives of the barriers they face [15, 18–20]. Finally, most existing research has focused on a single barrier at a time (e.g., distance from home to treatment, cost of treatment) [18, 21]. Though cumulative barrier models have been used to assess barriers to accessing substance use treatment [32, 33], they have not yet been used to measure barriers to methadone treatment retention.

The purpose of the current study is to (a) examine barriers to retention in treatment among patients receiving methadone at an OTP in a small urban community serving surrounding rural areas and (b) identify factors associated with greater cumulative barriers. Once rural-specific barriers to treatment retention are better understood, efforts can be made to mitigate them, ultimately improving treatment outcomes and reducing the impact of the opioid overdose crisis in rural communities.

## Methods

Data were collected at an OTP located in Jackson, Michigan, a small urban community in the Midwest US. The population of Jackson is less than 33,000 people, the majority of which are White (71.3%) [34]. The median household income is below, and the unemployment rate is above the state average [34]. The agency serves as a clinical hub for patients from several surrounding rural areas or “spokes,” as the closest methadone treatment provider for residents of three rural counties directly south of the clinic and rural census tracts to the north and west. In Michigan, most opioid treatment providers are concentrated in large urban counties and considerably fewer are in small urban or rural counties. Large urban counties average 3.50 OTPs, while small urban counties average 0.88 OTPs, and rural counties average 0.05 OTPs [35].

Data collection occurred over a three-week period in December 2019. In the weeks prior to data collection, OTP staff informed patients of the study and distributed study flyers. Research staff were present onsite for data collection three varied days each week. Interested patients (N = 267) were escorted to a private room onsite where they self-administered a computer-based survey. Research assistants obtained informed consent and assisted participants who requested accommodations due to reading, eyesight, or technology difficulties (n = 16, 6.0% of study participants). Surveys assessed demographics, substance use history, mental health, trauma history and symptoms, social support, and barriers to participation in treatment. Participants were compensated with a $20 grocery store gift card. All study procedures were approved by the Wayne State University institutional review board.

### Measures

Age (in years), gender (male, female, or other), racial/ethnic identity (White, African American/Black, Native American, Hawaiian/Pacific Islander, Asian, Arabic/Middle Eastern, Hispanic/Latino, or multiracial), and completion of high school or equivalent (GED) were included as demographic variables. The gender item included options for male, female, and other, though no participants reported a gender other than male or female. Because most participants identified as White (85.1%) and other racial and ethnic groups were small (≤ 5%), race/ethnicity was coded as White (Non-Hispanic) or Person of Color. To identify participants residing in rural communities, Federal Office of Rural Health Policy classification codes were used to classify zip codes of current residence as urban or rural [36]. This definition uses Rural–Urban Commuting Area (RUCA) codes and Rural–Urban Continuum Codes (RUCCs) to identify residents of rural census tracts and counties [36].

Participants reported any past year use of opioids (e.g., heroin, fentanyl, or non-prescribed opioid pain relievers) that were not for the purposes of medical treatment. Though participants could also report past year use of other drugs (e.g., alcohol, cannabis, stimulants, sedatives), the current study focused on non-medical opioid use only. Any past-year opioid misuse was then compared with the date that the participant started treatment to indicate the occurrence of past-year opioid misuse (a) prior to starting treatment or (b) while in treatment. Mental health symptoms were assessed using the Patient Health Questionnaire for Depression and Anxiety (PHQ-4) [37]. Participants indicated how often they had experienced four common symptoms of depression and anxiety in the past two weeks. Responses to the four items were scored on a four-point Likert scale from not at all (= 0) to nearly every day (= 3) and summed, with higher scores indicating more severe symptomology (Cronbach α = 0.90) [37]. Trauma symptoms were assessed using the Primary Care PTSD Screen for DSM-5 (PC-PTSD-5), a five-item scale predicting post-traumatic stress disorder (PTSD) based on major symptoms [38]. Respondents were presented with a definition of traumatic events, and those who indicated a trauma history responded to five items assessing symptoms of PTSD (Cronbach α = 0.89). Following the approach of Prins et al. [38], participants who endorsed at least three of the five items were classified as meeting criteria for probable PTSD. Level of social support was measured using the Social Support for Recovery Scale [39]. Respondents indicated their agreement with nine statements regarding how supported they feel in their recovery, including, “the people in my life understand that I am working on myself” and “the people in my life go out of their way to show me support.” Responses range from strongly disagree (= 1) to strongly agree (= 4). Four statements were reverse-scored, then the nine items were summed for a total score. Scores ranged from nine to 36, with higher scores indicating higher levels of social support (Cronbach α = 0.84).

#### Retention barrier inventory

Drawing on previous research [7, 10, 11] and feedback from the OTP staff, nine items were developed for the current study to capture barriers that may impact patients’ ability to participate in treatment. The barrier inventory examines the impact of the following barriers: childcare responsibilities, work schedule, housing instability, lack of reliable transportation, distance from home to treatment, legal obligations, treatment costs, mental health issues, and physical health needs. Reflecting on their general experience in treatment, participants indicated their agreement that each barrier has made it difficult to participate. Response options were provided on a four-point Likert scale from strongly disagree (= 1) to strongly agree (= 4). Items were summed for a total score, ranging nine to 36, with higher scores indicating greater cumulative barriers (Cronbach α = 0.87).

### Statistical procedures

Analyses were conducted using Mplus (Version 8.4), using full-information maximum likelihood estimation (FIML) to handle missing data. Study variables had 2.74% missing cases on average (Little’s MCAR test *χ*^2^(120) = 151.339, *p* = 0.028) [40]. Data were screened for assumptions required of linear regression analyses. Variance Inflation Factor values ranged 1.02 to 1.23, suggesting multicollinearity is not likely to be a problem. Descriptive statistics were calculated for all variables and t-tests were used to examine differences in individual and cumulative barriers by community type. Multiple regression was used to identify demographic and psychosocial factors associated with greater endorsement of treatment participation barriers.

## Results

Descriptive statistics for the sample are provided in Table 1. Over half of the sample was female (58.8%). Ages ranged from 22 to 72, with a mean age of 38.51 (SD = 9.91). The sample was largely White (85.1%), and the majority had earned at least a high school diploma or GED (77.5%). Fourteen percent resided in a rural community outside of the immediate area where the agency is located. The majority had been in treatment at the OTP for at least one year (80.3%). Just over half of respondents (54.2%) reported no past year opioid misuse. A small number of those reporting no past year opioid misuse had been in treatment at the OTP for less than one year (n = 4, 3.0% of those with no past year opioid misuse), likely representing patients who transitioned to the OTP from another area or another form of treatment (e.g., buprenorphine). Of the total sample, 13.4% of patients reported past year opioid misuse prior to starting treatment, and 32.4% reported past year opioid misuse while in treatment (i.e., past year return to use). Mental health symptom severity scores ranged from zero to 12, averaging 5.25 (SD = 3.75). Nearly half of the sample (45.1%) screened positive for PTSD. Respondents reported a range of social support experiences, with scores ranging from 10 to 36, averaging 25.37 (SD = 5.31).

**Table 1:** Sample characteristics (N = 267)

	*% (n)*	*M (SD)*
Gender		
Female	59.9% (157)	
Male	40.1% (105)	
Age		38.51 (9.91)
Race		
White	85.1% (222)	
Person of Color	14.9% (39)	
HS diploma/GED or higher	77.5% (196)	
Community type		
Rural	14.6% (37)	
Urban	85.4% (216)	
Past year opioid misuse		
None	54.2% (136)	
Prior to starting treatment	13.4% (34)	
While in treatment	32.4% (83)	
Depression/anxiety symptom severity		5.25 (3.75)
Probable PTSD diagnosis	45.1% (119)	
Social support		25.37 (5.31)

The majority (68.9%) of participants agreed or strongly agreed that at least one barrier made it difficult to participate in treatment. Over half (53.6%) agreed or strongly agreed that multiple barriers made it difficult. As shown in Table 2, lack of reliable transportation (M = 2.12, SD = 1.01), distance from home to treatment (M = 2.10, SD = 1.05), and work schedule conflicts (M = 2.09, SD = 1.02) emerged as the most highly endorsed barriers. Retention barrier inventory scores ranged from nine to 35, with a mean score of 17.02 (SD = 5.89). Compared to patients residing in the small urban community where the OTP is located, patients residing in surrounding rural communities reported greater difficulties related to distance from home to treatment (p < 0.001). No other significant differences in retention barriers were found by community type.

**Table 2:** Individual and cumulative barriers to participation in treatment among patients living in rural and small urban communities

	Full sample		Rural			Small urban			
	*% (n)*	*M (SD)*	*% (n)*	*M (SD)*		*% (n)*	*M (SD)*	*t*	*p*
Individual barriers to participation in treatment^a^									
Child care responsibilities	17.2 (45)	1.71 (0.86)	13.5 (5)	1.60 (0.85)		16.4 (35)	1.69 (0.81)	0.634	0.526
Work schedule conflicts	35.0 (90)	2.09 (1.02)	35.4 (13)	2.06 (1.00)		34.5 (72)	2.07 (0.99)	0.086	0.932
Housing instability	21.9 (57)	1.82 (0.90)	18.9 (7)	1.65 (0.78)		22.3 (47)	1.84 (0.91)	1.293	0.196
No reliable transportation	33.9 (87)	2.12 (1.01)	41.3 (15)	2.22 (0.95)		30.6 (65)	2.09 (0.99)	− 0.790	0.430
Distance from home to treatment	33.6 (87)	2.10 (1.05)	60.7 (22)	2.72 (0.99)		28.2 (60)	0.98 (0.99)	− 3.711	** < 0.001**
Legal obligations	15.5 (40)	1.66 (0.84)	8.1 (3)	1.43 (0.72)		16.3 (34)	1.69 (0.83)	1.874	0.061
Un/underinsured	19.2 (50)	1.79 (0.93)	18.9 (7)	1.73 (0.95)		18.3 (39)	1.79 (0.91)	0.352	0.725
Mental health needs	22.6 (58)	1.88 (0.91)	18.9 (7)	1.68 (0.77)		22.1 (47)	1.90 (0.93)	1.560	0.119
Physical health needs	18.7 (48)	1.80 (0.85)	21.6 (8)	1.70 (0.80)		17.7 (37)	1.81 (0.86)	0.764	0.445
Cumulative barriers to participation in treatment		17.02 (5.89)		16.86 (4.88)			16.85 (5.95)	− 0.014	0.989

^a^Individual barriers were rated on a four-point scale from strongly disagree (= 1) to strongly agree (= 4)

The frequency and percent of participants who agreed or strongly agreed that each barrier made it difficult to participate in treatment is presented beside the mean and standard deviation for each item

Multiple regression was used to assess demographic and psychosocial factors associated with cumulative barriers to treatment participation. Results are summarized in Table 3. The overall model was significant [*χ*^2^(10) = 29.379, *p* < 0.001], with an R^2^ of 0.155. Past year opioid misuse, depression and anxiety symptomology, and social support level contributed to the model significantly. Past-year opioid misuse while in treatment was associated with greater endorsement of barriers; past-year opioid misuse while in treatment was associated with a 2.31 unit increase in retention barrier scores (*B* = 2.311, *p* = 0.004). More severe mental health symptomology was associated with greater endorsement of barriers; every one unit increase in mental health symptom severity was associated with a 0.20 unit increase in retention barrier scores (*B* = 0.202, *p* = 0.038). Greater social support was associated with lower endorsement of barriers; every one unit increase in social support was associated with a 0.23 unit decrease in retention barrier scores (*B* = − 0.226, *p* < 0.001). No associations were found for demographic variables.

**Table 3:** Multiple linear regression analysis predicting cumulative barriers to participation in treatment

	*B*^a^	*SE*	95% CI	*p*
Age	− 0.023	0.038	− 0.086– 0.040	0.552
Gender: female	− 0.919	0.696	− 2.065–0.226	0.187
Race: white	− 0.409	1.087	− 2.197–1.378	0.706
HS diploma/GED or higher	0.376	0.816	− 0.967–1.719	0.645
Community: Rural	− 0.026	0.890	− 1.490–1.438	0.977
Past year opioid misuse^b^				
Prior to starting treatment	0.853	1.041	− 0.858–2.565	0.412
While in treatment	2.311	0.800	0.995–3.627	**0.004**
Depression/anxiety symptom severity	0.202	0.097	0.042–0.362	**0.038**
Probable PTSD diagnosis	0.715	0.781	− 0.570−1.001	0.360
Social support	− 0.226	0.064	− 0.332 to − 0.121	** < 0.001**

^a^*B* unstandardized coefficients

^b^No past year opioid misuse is reference group

## Discussion

This study adds to the limited knowledge of barriers to retention in methadone treatment in small communities. This line of research is needed to guide efforts to improve the accessibility and quality of OUD treatment. Prior research has shown a positive association between treatment duration and substance use outcomes [41, 42]. Patients who perceive greater barriers to participation in treatment, particularly those who experience multiple barriers, may be vulnerable to return to use and premature discharge. Examining each of the retention barriers individually, endorsement of barriers in this sample appears fairly low (i.e., no more than one-third endorsed a single item). Considering the full range of barriers, however, most patients endorsed at least one barrier, and more than half endorsed multiple barriers to retention in treatment. This finding highlights the value of the novel cumulative barrier approach presented here. Future research should consider a broad range of barriers to retention in treatment, as the challenges patients face are numerous and varied. Efforts to address these barriers, such as flexible regulations for dispensing methadone, co-location or care coordination, and family or peer support programs, may more effectively reduce opioid use and related harms in small communities.

In the current study, travel hardships (i.e., distance from home to treatment, lack of reliable transportation) and work schedules were the most frequently endorsed barriers to participation in methadone treatment, with about one-third of participants reporting such barriers. These results are consistent with a systematic review which identified travel hardships related to seeking care from distant providers (e.g., further distance, longer travel, cross-state commute) as the most common accessibility barriers for rural people with OUD [8]. Transportation services such as mileage reimbursement [43] and nonemergency medical transportation (e.g., Lyft, Uber) [44, 45] have improved treatment access and retention in other health care settings, and may reduce transportation barriers for rural methadone patients. These programs provide patients at or above a designated travel burden threshold with mileage reimbursement or travel vouchers. Such initiatives have recently been implemented at OTPs, with anecdotal reports of broad utilization and patient satisfaction [46].

Travel hardships are exacerbated by legal and regulatory barriers for methadone, which require most patients to visit treatment programs daily. These requirements can be time consuming and impede patients’ ability to obtain and maintain employment [47]. Policies that lift legal and regulatory barriers to prescribing methadone in mainstream health care settings may improve the availability and accessibility of methadone, thereby reducing travel and work-related barriers to participation in treatment. A shift to a pharmacy dispensing model, for example, could reduce travel time by nearly 20 min (one-way) for patients in small urban communities and as much as 51 min (one-way) for patients in rural areas [48], mitigating geographic disparities in methadone access [49]. Pharmacy settings may also offer longer or more flexible hours than OTPs, further reducing time burden and schedule conflicts. Alternatively, methadone could be delivered to patients at their private residences. Mobile medication programs have been shown to increase treatment participation and retention, particularly among high severity, underserved groups of persons with OUD [50, 51]. In March 2020, in response to COVID-19, the US Department of Health and Human Services provided OTP guidance to permit home delivery of methadone, expand take-home privileges, and allow a trusted friend or family member to pick up medication [52, 53]. These changes have allowed patients more flexibility to work or engage in other productive activities [54], and have resulted in very little diversion [55]. Many advocates call for flexible dosing and delivery options to continue once the pandemic is resolved [56]. By increasing the availability and accessibility of methadone and reducing time and travel burden, pharmacy models, home delivery, and flexible dosing options may effectively reduce work-related barriers to treatment participation.

Though previous studies have found differences in OUD treatment access and retention based on gender, race/ethnicity, and age [10, 15, 16, 19], we found no association between demographic variables and cumulative barriers. These discrepancies may reflect differences in measures and community type. Previous studies have measured individual barriers and have not focused on patients in less populated areas. Some demographic differences may be less pronounced in smaller communities which tend to be less racially diverse. It is notable that no significant association was found between residence in a nearby rural community and cumulative barriers to treatment. In the current study, data were collected in a small urban community that is just below the federal population cut point for rural designation.^1^ Thus, the small urban and rural communities included in the sample are similar, and residents appear to face similar retention barriers. Results of bivariate analyses indicate living in a rural community may be more strongly associated with travel barriers related to distance from home to treatment than with the other barriers included in the cumulative barrier measure (e.g., housing instability, mental health needs). While patients residing near the OTP in the small urban community may therefore experience fewer travel barriers, our findings suggest rural and small urban residents in this sample experienced a similar degree of cumulative barriers overall. It is also possible that for individuals living in a rural community, barriers have a more substantial effect on treatment initiation than they do on retention in treatment. Future studies should compare retention barriers in rural settings to those in large urban areas and examine the cumulation of barriers that prevent rural residents from initiating methadone treatment.

Past year opioid misuse while in treatment was associated with greater endorsement of barriers, highlighting the time following return to use as critical for the provision of wraparound services and support. This may be particularly important for individuals with OUD, who report fewer resources and less support than individuals with other substance use disorders [57]. Given the cross-sectional nature of the current study, the temporal relationship between return to use and cumulative barriers cannot be discerned. It could be that the consequences of return to use create barriers to participation in treatment. This could include, for example, consequences of criminal legal system involvement related to return to use (e.g., loss of driver’s license, court fees, community service), or erosion of social support from family members or others in recovery. Such consequences could make participation in treatment more difficult. It is also possible those with greater cumulative barriers are more vulnerable to return to use. We suspect a bidirectional relationship, in which return to use leads to greater barriers, and greater barriers increase vulnerability to return to use. Further research is needed to disentangle the relationship between return to use and barriers to participation in treatment.

More severe mental health symptomology was associated with greater cumulative barriers, suggesting patients experiencing symptoms of depression and anxiety may be at risk for discontinuation of treatment. Though the causal mechanisms that link mental health to treatment barriers are not indicated, it is clear that those with symptoms of anxiety and depression may benefit from additional support. For patients receiving methadone, identification of co-occurring mental health conditions should be prioritized, and linkage to community resources (e.g., government benefits) may be offered alongside evidence-based integrated mental health and OUD treatment. Co-located psychiatric services may further reduce barriers and improve outcomes for patients with mental health issues.

Greater levels of social support were associated with fewer barriers to participation in treatment, highlighting social support as a protective factor. This finding is consistent with a large body of research which demonstrates a positive effect of social support on long-term recovery [24–26]. Social support has been identified as important across three specific levels: concrete, emotional, and informational [58]. For this population, concrete support might entail transporting the patient to and from the clinic, assisting with child care, or providing safe, substance-free housing. At an emotional level, support may facilitate encouragement to continue in treatment when the individual feels discouraged [28, 30]. Informational support could include helping patients access government benefits to assist with child care, housing, treatment, or transportation costs. As such, increased family and peer support may improve treatment access and retention. Findings from the current study provide support for interventions that build and strengthen support networks for methadone patients, such as family programming and integration of peer staff.

### Limitations and implications for future research

Results should be interpreted in consideration of study limitations. Data for this study were collected cross-sectionally from patients receiving methadone at one agency located in a small urban community in the Midwest US. It is not possible to make directional or causal interpretations of findings. Data collection took place over three weeks, with research assistants onsite three varied days each week. While some patients visit the agency daily, those who have been in treatment longer may visit the agency as little as once every other week. As such, the current sample may overrepresent patients in earlier stages of treatment who visited the agency more often. Some patients reported that time constraints due to transportation, work, or child care made it difficult to participate in the study. Many were able to arrange to complete the survey while research assistants were onsite, but some may have been unable to do so. Patients who were not present during data collection and those who could not participate due to time constraints may differ from study participants regarding barriers to treatment participation. Though the OTP was the closest methadone provider for several surrounding rural communities, only a small portion of study participants (14.6%) were living in areas designated rural. Because few OTPs exist outside of large urban areas, assessing barriers to retention in methadone treatment among rural residents is challenging. Though rural and small urban residents in the current study did not differ significantly by cumulative barriers, future studies should consider different recruitment strategies to assess retention barriers in predominately rural samples. This could include, for example, calling or mailing surveys to rural patients served by multiple small urban OTPs. Additionally, while some patients reported past year use of other substances, the current study assessed past year return to non-medical opioid use only. Future studies should examine the association between comorbid SUDs and cumulative barriers to methadone treatment. It should also be noted that the current study focused on the experiences of individuals in OUD treatment; these individuals may logically face fewer barriers than those who experience barriers that prevent treatment initiation. Further research is needed to understand the cumulation of barriers that prevent individuals from initiating methadone and other medications for OUD in small urban and rural communities. Finally, the self-report data presented may be limited by recall and social desirability biases. For example, some participants may be hesitant to label something as a barrier and may consequently underreport factors that impact their ability to participate in treatment. Future research may consider more nuanced multi-item measures of barriers to participation in treatment.

Despite these limitations, findings from the current study provide novel and valuable information about barriers to retention in methadone treatment in a small urban community, while suggesting areas for future research. Replication of the current study in other geographic locations or with individuals receiving different forms of treatment for OUD may reveal differences in barriers to participation in treatment across populations and settings. Measures of treatment barriers that assess internal processes such as privacy concerns or self-stigma [32] may yield further insights that could help improve substance use treatment in rural and small urban communities. Future studies may also assess causal mechanisms which link the identified risk and protective factors to perceived barriers. Studies that explore the interrelated nature of treatment barriers may point to where future interventions may focus to reduce cumulative barriers to treatment. For example, efforts to reduce the distance from home to treatment may help alleviate conflicts related to work, child care, and legal obligations. Given that endorsement of individuals barriers was relatively low, qualitative studies should explore the strategies patients use to overcome barriers to participation in treatment. Findings may illuminate practical approaches to improving treatment retention that have not yet been explored. Finally, longitudinal studies should examine the impact of individual and cumulative barriers on treatment and recovery outcomes (i.e., do retention barriers predict substance misuse and treatment retention at one-year follow-up). Identifying the individual barriers with the strongest effect on treatment retention may point to critical areas for intervention and help guide the development of more advanced, weighted cumulative barrier measures.

## Conclusion

Adults in this small Midwest community report a number of barriers to participation in methadone treatment. Identifying treatment barriers and risk factors for cumulative barriers is important for improving treatment retention and recovery outcomes. Findings suggest those with past-year return to use and those with co-occurring mental health issues face greater barriers to treatment retention. Efforts to reduce time and travel burden and expand or strengthen social support systems may improve patient experiences and treatment outcomes.

## Data Availability

Data from this study are available on request from the corresponding author (EP).

## Abbreviations

OUD: Opioid use disorder
OTP: Opioid treatment program
RUCC: Rural–Urban Continuum Code

## Footnotes

1 Jackson, Michigan has a Rural–urban Continuum Code (RUCC) of three; RUCCs one through three are designated metropolitan (i.e. urban) and RUCCs four through nine are designated nonmetropolitan (i.e. rural).
